# Making sense of Heidegger’s ‘phenomenology of the inconspicuous’ or inapparent (*Phänomenologie des Unscheinbaren*)

**DOI:** 10.1007/s11007-017-9422-8

**Published:** 2017-06-22

**Authors:** Jason W. Alvis

**Affiliations:** University of Vienna, Vienna, Austria

## Abstract

In Heidegger’s last seminar, which was in Zähringen in 1973, he introduces what he called a “phenomenology of the inconspicuous” (*Phänomenologie des Unscheinbaren*). Despite scholars’ occasional references to this “approach” over the last 40 years, this approach of Heidegger’s has gone largely under investigated in secondary literature. This article introduces three different, although not necessarily conflicting ways in which these sparse references to inconspicuousness can be interpreted: (1) The a priori of appearance can never be brought *to manifestation*, and the *unscheinbar* (inconspicuous) is interwoven with the scheinbar (appearing) as an active characteristic or form of “hiddenness” (Λήθη), therefore making inconspicuousness inherent within all phenomenology. (2) Or, there is now a particular step or reduction within phenomenology that involves one’s being attuned to the various modes of potential hiddenness (Verborgenheit and its cognates), of which “inconspicuousness” is a particular character trait. Or (3) there are particular, unique, and specific phenomena that give themselves “inconspicuously,” and there is also thus a corresponding, particular phenomenology in which one must engage in order to gain some kind of access to these specific things’ phenomenal strata. This paper introduces Heidegger’s “phenomenology of the inconspicuous” most especially in his last seminar in Zähringen in 1973, engages related references to unscheinbar in his 1942/1943 Seminar on Parmenides, and then puts forward an interpretation of what these somewhat ambiguous references could mean when contextualized according to Heidegger’s overall interests. This essay brings these references to light, and puts forward a proposal as to what kind of phenomenology Heidegger was–somewhat inconspicuously–referring.

The essence of genius is to know what to overlook—William James.

A Phenomenology of the “Inconspicuous” is a means of investigating and subsequently experiencing (without “conceiving”) phenomena whose intelligibilities oscillate between both presence and absence. This, at least is a starting point for how Heidegger’s reference to such a “concept” might be understood. For this reason, and for others, it plays a role in the formation of a “para-doxical” (as “contrary to appearances”) provenance that gets beyond the dialectic Heidegger once called “a genuine philosophical embarrassment,” by reaching down to the fundament of what makes the dialectic between appearance/non-appearance possible, as absence “presents” itself. Although the German adjective unscheinbar (inconspicuous) could be translated directly as the privation of appearance, as “non-shining,” or as in-sign-ificant, it perhaps more specifically characterizes what resists providing something meaningful through signification, yet still furnishes an intelligibility with which we more implicitly are “involved.”

What is unscheinbar is not brilliant (glänzend), bright (leuchtend), apparent (offenbar), or clear (klar), yet in every case it never should be confused with being invisible (unsichtbar). “Inconspicuousness” amounts to a fundamental experience with the unobtrusive (unauffällig) yet in a way that makes no special impressions upon us. un-schein-bar draws from its root Schein, which today commonly is used to refer to a license, ticket, or warrant, phenomena characterized by being unquestionably trustworthy (e.g. Der Fahrschein, a travel ticket). Yet what is scheinbar has a slightly different sense, referring not to that which is claimed to be obvious, but rather to what one openly surmises or conjectures to be true. Thus, what is Unscheinbar resists even one’s attempt to estimate or properly investigate its status; it obscures even the possibility of “seeming.” This is perhaps one reason why unscheinbar has taken on the more everyday, colloquial reference to the “wallflower,” which is fully present, yet absent to conscious awareness. Thus, for a phenomenon ontologically to be unscheinbar it would need to not-be, while still maintain the status of being inconspicuous or “inapparent.”

The English and French “inapparent” generally has been used to refer to symptoms that go unnoticed, such as viruses that exist, yet do not show themselves in any direct way. As the inverse of the “apparent” or “obvious,” (with its origins in the Latin verb apparere), it is what does not appear and remains latent or dormant. Yet “inapparency” does not quite capture the full meaning of unscheinbar in the way the word “inconspicuous” does. Inconspicuousness characterizes that which does not easily give or present itself and–again like the “wallflower”–is not readily noticed on the account of not drawing or grabbing attention. It can be traced to the 15th century Latin inconspicuous, which refers to what is not “striking” or specere as a known and celebrated spectacle.^1^ This is one reason why the present study prefers to translate unscheinbar as “inconspicuous” as opposed to “inapparent.”^2^

Aspects of such an etymology likely contributed to Heidegger’s somewhat elusive description of his “phenomenology of the inconspicuous” (Phänomenologie des unscheinbaren), a phrase he did not even utter until his last seminar, which was in Zähringen in 1973.^3^ Such elusiveness, which has been compounded by a lack of consensus on how to translate the unscheinbar into English) have left little room for clarifying what appears to be a sparsely referenced, although fecund provocation of a method that gains its namesake from its interest in dealing strictly with shining, “apparent” things through its enterprise of clarifying and bringing into description the various strata of how phenomena appear. Thus, any mention of a phenomenology of the inconspicuous elicits a challenge to the basic principles of phenomenology in toto and calls for a certain revision of the pretensions of how consciousness constitutes its world and engages in the reflective “pure inwardness” of intentionality–an active basis of Husserl’s transcendental-phenomenology. Under the pretensions of the latter, phenomenology can be employed only to offer reductions of intelligible things insofar as they are in and of this world. As most Heidegger scholars will concede, such an appearing/non-appearing dialectic is precisely what Heidegger sought to dispel throughout his lifetime, most especially in his critique of the metaphysics of presence, which amounted to the ignorance of the inter-sutured nature of presencing (Anwesen, typically understood as the absent, hidden, or to-be-presented) and presence itself (beständige Anwesenheit).^4^ Jean-François Courtine argued that even within Sein und Zeit there is already an active embrace of a phenomenology of the inconspicuous in its emphasis upon the facets and strata of covering and “uncovering” (désoccultation). Nothing can be taken for granted, not even the conscious “subject” for whom the world is rendered phenomenally visible. Thus, a phenomenology of the inconspicuous, with its vague contours and deep relations to the various modes or “types” of concealment, stands as a challenge to, and transgression of not only any preference for the visible, but also its seemingly opposite–the invisible or “not present.” How far this approach can remain phenomenology “as such” is yet to be determined.

In 1991 Dominique Janicaud explicated some of the consequences of such an approach when he critiqued the so-called “Theological Turn” in French Phenomenology for its reliance upon a certain sick root within phenomenology: Heidegger’s phenomenology of the inconspicuous (unscheinbaren).^5^ Janicaud interpreted these thinkers in their misguided “approach” to open up phenomenology to new intimacies with the invisible and that which does not appear within our subjective world of experience (Erfahrungswelt) instead of focusing on things that are obvious (selbstverständlich) and immediately given. The approach that was once accorded by Husserl to be “methodologically atheistic” was being “hijacked” by these French theologians in disguise. Yet Janicaud never closely clarified just what Heidegger really meant when he referred to such an approach, and how it might be contextualized in his oeuvre, despite a minimal engagement with the topic as it relates with temporality in Janicaud’s Chronos.^6^ Instead, Janicaud appeared to conflate incorrectly the terms unsichtbar (invisible) and unscheinbar (inconspicuous) despite his attunement to how this concept of Heidegger’s is largely significant for bringing thinking to attention to what in “appearance does not appear.”^7^ Janicaud instead interprets Husserl’s approach to focus soley on the “radiance” of things as they show themselves (phainesthai).^8^

Since Janicaud’s minor treatment of Heidegger’s phenomenology of the inconspicuous, most interpretations have taken shape similarly, and it therefore has gone without receiving a careful treatment or assessment.^9^ For example, Günter Figal’s recently published Unscheinbarkeit (2015) is a shining example of how inconspicuousness can be employed as a phenomenological concept. Figal names inconspicuousness to be the primary characteristic of “space” (Raum), which is the fundament of that which appears and its possibility for being given, thus changing phenomenology itself.^10^ Yet despite clearly being inspired by Heidegger’s work in these regards (Figal even suggests that he was led to the question of space through Heidegger’s Freiraum or openness), Figal only very briefly engages Heidegger’s treatment of the concept, and instead remains devoted to his more constructive development in regards to space. Although Figal recognizes that Heidegger was the one who initiated inconspicuousness into phenomenological thinking (“Dass es Phänomenologie, die von Unscheinbaren her denkt, bisher noch nicht gab, könnte eine voreilige Behauptung sein… Heidegger hat ihn geprägt”^11^), Figal ultimately suggests that Heidegger’s “tautologisches Denken” (tautological thinking) is not “wirklich eine Phänomenologie des unscheinbaren” (not really a phenomenology of the inconspicuous).^12^ For these reasons, and for others, a full treatment of Heidegger’s position was not the goal of Figal’s recent book.

This merely highlights how there are a number of questions remaining to be posed concerning Heidegger’s thinking on the inconspicuous. Is such an approach indeed a potential threat to phenomenology, which relies upon the visible, as Janicaud concludes? And does this approach necessarily entail a disposition to religious or metaphysical experience? Further, does this concept play any impactful role in the (especially late) thought of Heidegger’s, and if so how might one negotiate the somewhat ambiguous usages of the term “phenomenology of the inconspicuous” in one seminar, Heidegger’s last, no less? This essay makes three steps in understanding the directions Heidegger could be going with this concept. The essay offers a contextualization of the concept in the framework of the Zähringen Seminar, an interpretation of his usages of the term in the Parmenides Seminars, and then a determination as to what the most likely interpretations of this concept might be, given Heidegger’s overall interests (which never are without some controversy). It is argued here that there are three different ways in which one might treat these references, which all point to a particular “approach” to the inconspicuous: (1) Following Heidegger’s reformation of Husserlian phenomenology according to how the a priori of appearance can never be brought to full clarity, Unscheinbarkeit is marked by its being interwoven and integrated within “hiddenness” (Λήθη, lethe) that allows things be brought into presence; it is germane to the “clearing” (Lichtung) and interwoven within every aspect of appropriated human existence (ereignete Dasein). This would entail that inconspicuousness is inherent within all of Heidegger’s phenomenology. (2) Another option is that Heidegger is introducing a particular step into all of phenomenology that involves ones being attuned to the various, potential modes of hiddenness (Verborgenheit and its cognates) within all phenomena, and “inconspicuousness” is now to be included as a form, mode, or “manifestation” among them.

(3) Or, there are particular, unique, and specific phenomena that have a greater tendency than others to give themselves “inconspicuously,” and if so, it is likely that there is also a corresponding, particular phenomenology in which one must engage in order to access their intelligibility. Such an approach might allow further access to the site of interaction or touch-line between the potentially multivalent forms of “withdrawal” and presence (das Anwesen), which take place in presencing (die Anwesenheit).^13^ Although there is a level of ambiguity (even to the point of seeming sophistical) to Heidegger’s references to unscheinbar in the Zähringen Seminar, I would suggest that they can play be contextualized, and play an important role within how we are to understand the broader interests of Heidegger concerning the status of Da-sein, the “how” of appearance, and the grounding of phenomenology in relation to “the clearing” that to some degree is metonymic with Being.^14^ Further, his occasional uses of the word unscheinbar, namely in his engagements with Parmenides, help illuminate a red thread that runs throughout these works, thus offering a greater degree of precision in an interpretation of the concept.^15^

## Inconspicuousness in the 1973 Zähringen seminar

Often inspired by an interest in synthesizing Heraclitus’ emphasis on becoming with Parminides’ ontological “it is,” Heidegger emphasized how, as Dreyfus put it, there is a uniquely “saving power of insignificant things,” namely those that resist technological “machination.”^16^ Things that hold strata of ordinariness are not necessarily “insignificant” (as some have translated unscheinbar) in the sense that they utterly lack a significatory process, but rather simply do not draw immediate attention within conscious experience. For something to be “insignificant” in these terms is for such a thing actually to bear great significance, despite its having certain tendencies or traits of being easily overlooked. The most explicit attention paid by Heidegger to such inconspicuousness (as a concept at least) was in his Seminar on the outskirts of Freiburg in Zähringen, in 1973:

thus understood phenomenology is a path that leads away to come before…, and it lets that before which it is led show itself. This phenomenology is a phenomenology of the inapparent [unscheinbar]. Only now can one understand that there were no concepts for the Greeks. Indeed, in conceiving [Be-greifen], there is the gesture of taking possession. The Greek … on the contrary surrounds firmly and delicately [that] that which sight takes into view, it does not conceive.^17^

To understand such a phenomenology, it first must be acknowledged that it is tautological and paradoxical. One must follow in a “way” (weg) of thinking whereby one engages in how distance creates nearness (der hinführt vor…und sich das zeigen läßt), and how this distance or “awayness” is the only means by which one can experience the thing as it shows or bears its intelligibility. This (diese, that is, something particular) phenomenology is “a phenomenology of the inconspicuous.”^18^ “Away” and “before,” modes of distance and closeness that are basic forms of relation, play a formative role in the experiencing of the inconspicuousness of phenomena.

Throughout the Zähringen seminar Heidegger alludes, in a final reflection almost 45 years later, back to what he thinks to be the original merit of Sein und Zeit. The seminar is propelled by a question posed by Jean Beaufret that leads Heidegger to reflect upon the uniqueness of his life work, as it is to be distinguished from Husserl’s: How and why did Heidegger find it necessary to turn from Husserl’s method? In answering this question, Heidegger alludes to how such a turn was established on the basis of Husserl’s negligence of the ever-important, non-metaphysical “meaning” or truth of being. “Being in the world,” Dasein (Ex-sistence, being-open) is a more originary experience to thinking than a being-conscious (Be-greifen, conceiving), and one can somehow access it through what we constantly take for granted: the ever-hiding and concealed “clearing” work of Being. Although Husserl makes reference to “being” as one of the means of relation in consciousness, he never addresses or inquires into its meaning in and of itself, and simply follows with a rudimentary understanding of Being (das Sein) as a “constant, steadfast presence” [Anwesenheit und Beständigkeit], thus overlooking the appropriation of ex-sistence. Yet at the same time, Being (and our experience of it in/as the “clearing”) is fundamentally hidden and withdrawn.^19^ Being is the basic way in which things reveal themselves and their intelligibility. The truthing or disclosedness (dis-covery) of Being (which also entails various laminates of concealment), as Heidegger interprets Sein, holds the keys to one’s most fundamental experience with things. Such a truth is “always already” both temporally and spatially “before.” As he refers to ἀλήϑεια in SuZ, “unconcealment” has a “self evident” and “pre-philosophical basis.”^20^ Thus, the worldhood of the world is but a means to “raise anew” the question of being (and the clearing of Being), which in its pre-conscious and pre-philosophical truth (or clearing) is to be understood.

In starting to answer Beaufret’s question in the Zähringen Seminar, Heidegger references the differences between sensuous intuition and categorial intuition, and the ways in which one’s initial experience with objects, for example, is not with their sensual data, but actually with their categorial projections. Despite my material experience with brown wood and four posts, I only “see” the table because I am involved with it in particular ways, and therefore treat it as a “table” because it is pertinent to my involvement in the world. I take it as a table in order to toss my house keys on it. Thus the object’s coming into appearance is not first the result of sense data, but my particular representations of what it does for me in that moment. This leads to a perplexing paradox: Sense data and substantiality are what we most truly experience, yet we most often overlook them. The hyle, the building blocks of the senses (color, taste, shape), are what make objects’ manifestations possible, yet in preference for the thing we wish to see, the data they present elusively fall from view. Hyle become inconspicuous insofar as they are effective upon the changing structure of consciousness, yet in a way that they get overlooked due to their seeming banality. The fact that the hyle escape our notice in favor of what categorial intuition presents is not to be criticized as a result of our “falleness” per se, but is further proof that we are situated beings whose interests in things cannot be so easily distanced from our experience. Consciousness seems to prefer that which, in the past, has been visible or memorialized, over seeing that which does not so straightforwardly appear. This amounts to one preliminary meaning of inconspicuousness.

Yet there is another aspect of the categorial/sensuous relation upon which Heidegger calls us to reflect: Our relation with the categorial indeed is mediated, and this points to how the appearance of things has a tendency to make semi-permanent impressions that fix phenomena into place without the conscious movement of taking “things as.” To take something “as” entails the recognition that there is a certain unfixed relation one has with things and their various meanings, which one must “think” discursively (dis-currere) by traversing back and forth between one’s thoughts about the matter at hand, and the things that show themselves.^21^ As Tom Sheehan recently has conceived it, this is the ever-important process of “making sense” in Heidegger’s work. Yet Husserl, (especially in his conception of consciousness as Bewuß-sein) follows much of the rest of the philosophical tradition of metaphysics, makes the faulty preference for fixation, and therefore privileges that which appears over that which retains any obscure ambiguity.

There are, of course, many relations one has with what does not appear. Yet one typically relates with it according to the hope and interest in making its data come into appearance, and thus, such a relation is still centered around, or motivated by an interest in appearance. In other words, the preference remains for that which appears or is revealed over that which inconspicuously is hidden or obscure. Husserlian being-conscious is a matter of regarding, preserving, and safekeeping that which one has seen or known, beginning with a presuming ego cogito that not only prefers appearance and presence, but initially operates according to a number of pre-understood distinctions, such as appearing/non-appearing, inside/outside (though Husserl often explicitly rejects this distinction), and covered/uncovered. These matrixes of opposition effectively undermine the phenomenological project and its status as Erste Philosophie, for they reduce its interests and therefore thinking to a series of dialectics, which Heidegger vehemently called a “genuine philosophical embarrassment.”

Instead, Heideggerian Being is the lever that allows for one to go beyond both conceiving of the objecthood of the object and the distinction between the sensuous and categorial; and for relating with what we take to be seemingly ideal preferences for that which straightforwardly appears to us as obvious, and comes to be regarded by us as clear. Although unscheinbar is not referenced in Sein und Zeit, he does refer to cases of phenomena that do not directly give themselves or “shine,” but indeed still retain phenomenality: “This is what one is talking about when one speaks of the ‘symptoms of a disease,’” which is a sort of phenomenon that employs other phenomena as a proxy on their behalf. A Krankheitserscheinung is that “which does not show itself,” yet still “announces” itself as a disturbance in a healthy body.^22^ In such cases the phenomenal “experience” one has is with the symptom/indication itself, which represents what remains undisclosed. This points to how, claims Heidegger, a thing’s appearance must bear the marks of a “double signification: first, appearing, in the sense of announcing itself as not-showing-itself; and next, that which does the announcing [das Meldende selbst]—that which in its showing-itself indicates something which does not show itself.”^23^ As He continues, “phenomenon” should be understood in this third, more genuine sense of “appearing,” and as he crucially argued throughout his career, Sein belongs to, and displays itself within what shows itself and appears. Although phenomena never fully manifest themselves, this “not-showing” itself is a phenomenon.

Thus, there is a sense in which phenomenology must become the study of how one looks past things on both the sensual and categorial levels, and towards da-sein, ex-sistence or open-being, which gives space (Raumlichkeit) for both the sensual and categorial to appear. In SuZ, the thing is in the world, which is “not immanent” to consciousness; Dasein becomes the ek-statically “outside” and made-to-open (as Ereignet, appropriated) beyond the stationary and immobile.^24^ It is here in, and on the level of Being that “immanence is broken through and through” yet there is still a sense in which one relates with things instantaneously via a going “out of oneself.” One can be attuned to such a state of ex-sistence, as one is always already in this state of being-in-the-clearing outside of oneself. Such an attunement or relation is instantaneous, for one is in relation not with the mere presence-to a thing, but with presencing itself (Anwesen-heit). The “here and now” and the “sensical” are brought into presence and given sense. Even instantaneousness, the “immediate” (sofort) that lacks a medium or “go between,” is subject to the temporal and spatial dimensions of being-open. This is the necessary distance or space that makes up the comportment of Dasein.

This initiates a turn to the meaning of presence (or “meaningful presence”), which plays an indicative role in understanding that something is, or in taking something as, namely in this case, as inconspicuous. The present itself is given or brought into manifestation or presence. How, though, might the present presence itself? Could there be differing modalities or “laminates” of presence? Such modalities could be indicated, despite their not giving themselves in and of themselves in any straightforward way. The key, perhaps, is that one is to hold, for as long as possible, presencing into view by engaging the truthing disclosure and simultaneous closure of presencing, as they both creatively form presencing itself. The present is essentially “truth-being,” and in order for it to be accessed, one must follow a certain paradoxical path (the path that “leads away in order to come before”) in order to truly relate with that which is at home or before oneself.^25^ It is possible to conceive of two kinds presencing: a before-presence and an away-presence. No such kind of paradoxical away-presence was thought by Husserl, yet for Heidegger such absence or “awayness” is essential to attaining a phenomenology befitting of a human condition marked by constant projection beyond itself.

These general interpretations of Heidegger’s “inconspicuous” in relation to categorial intuition and presencing are confirmed further in a letter written to Roger Munier shortly after the seminar at Zähringen. There, Heidegger insinuates how his approach to categorial intuition is to be distinguished from Husserl’s (as found in the 2nd section “sense and understanding” in the 4th of the logical investigations). For Heidegger, it is about “actually performing an exercise in a phenomenology of the inapparent [unscheinbar],” which allows one to attain a “phenomenological ‘seeing.’” The use of the word “exercise” here something not unlike a “reduction” that would involve an investigation (that is to say a study of the how structure of the appearance) of that which is present-absent or “inconspicuous.” Heidegger continues in the letter, suggesting to its recipient that “you can easily link this text to what particularly concerned you [Munier] in my lecture ‘What is Called Thinking?.’”^26^ The essential element of “What is Called Thinking” that can be claimed to bear any marks of such an “exercise” in reaching the inconspicuous is in its outright description of the potential relations of the interplay between withdrawal and arrival, “before” and “away.” When one attempts to think or at least respond to the call of thinking, something strange happens:

That which is to be thought turns away from us. It withdraws from us. But how can we have the least knowledge of something that is withdrawn from the outset? How can we even give it a name? Whatever withdraws refuses arrival. But–withdrawing is not nothing. Withdrawal is event [appropriation, ereignis]. In fact, what withdraws may even concern and claim man more essentially than anything present that strikes and touches him.^27^

What is absent is not invisible, but rather is resisting actively the attempt to bring it into phenomenalization. This is related to withdrawal and inconspicuousness in Poetry Language Thought:

The inconspicuous [unscheinbar] thing withdraws itself from thought most stubbornly. Or can it be that this self-refusal of the mere thing, this self-contained refusal to be pushed around, belongs precisely to the essential nature of things?^28^

Again, withdrawal is not marked by a privation of sense, but by a positive ability to resist. And finally, of note in Unterwegs zur Sprache is the kind of relations we have with this withdrawal, which is the event/being appropriated:

Das Ereignis ist das Unscheinbarste des Unscheinbaren”–“appropriation is the most inconspicuous of the inconspicuous.^29^

“Things,” which are unscheinbar, inherently are elusive to our attempting to keep them from withdrawing. Withdrawal (Entzug) is equated with the appropriated or mine-made appropriation (Ereignis), which so impressively is inconspicuous. That is, the retreat of that which mobilized into the shadows of thought has a form (perhaps its very own) of impressing itself upon us, most especially as its movements are appropriated as withdrawn. Again, this is not withdrawal merely as invisibility (Unsichtbarkeit) or even as a form of mere hiddenness, but a withdrawal that “gives” (i.e., giving one the experience of its withdrawal and thereby being of concern to us) even in its achieving the status of moving away, and retreating from being before us in visibility (Sichtbarkeit). This should serve as a reminder that inconspicuousness must exceed the distinction between appearance/non-appearance.

## Concealment/unconcealment in the early Parmenides seminar

Before turning back to the Zähringen Seminar, it is helpful to contextualize the aforementioned interpretations of unscheinbar alongside other references in Heidegger’s work. The earliest, meaningful references Heidegger makes to the word unscheinbar (though no explicit “exercise” in experiencing it) is found in the Parmenides seminar in the winter semester of 1942/1943 (GA 54), in the context of Heidegger’s once again emphasizing how the false dialectic between unconcealedness and concealment should instead be thought according to distinct modes (Weisen). Our understandings of truth and concealedness far too often lead us to quarantine the sphere of mystery to the “merely not yet known.”^30^ We thus must return to the various cognates or intelligible meanings of concealment, which find their essential meanings in truth (ἀλήθεια, aletheia).^31^ For Parmenides “Being” in its most fundamental sense is ἀλήθεια. In order to experience the truth of truth (the privative “ἀ” of “λήθεια”), it is necessary to “be led,” according to Parmenides in Fragment 8 “to the ‘that it is’..” and for Heidegger, this amounts to being “out there” in the open, yet never straightforwardly accessible “clearing” or Lichtung (ZS p79).

In Heidegger’s seminars the dimension of lethe (Λήθη) is conceived as an a priori aspect of unconcealment as it already contains within itself laden aspects of concealment (e.g. withdrawal). Heidegger makes this explicit: “The essential form of unconcealment, [the active Unverborgenheit]…in a certain way retains within itself concealment [Verborgeneheit] and concealment [the passive Verbergung] and even must do so.”^32^ Some variations of concealment are at work always already in unconcealment in order for unconcealment to be properly unconcealment as such. Concealment (which he later also calls “disclosure,” or Entbergen) is essential to the make-up of unconcealment.^33^ How does this work and how might one “experience” it? Overall, there are two general ways in which we might interpret the operative functions of such concealment: First, there is concealment as such, which for “the Greeks the essence of concealment [Verbergung] and unconcealment [Unverborgenheit] was experienced so essentially as the basic feature of Being itself.”^34^ It is this concealment/unconcealment that operates as such a “primordial essence” that goes beyond any interpretation of concealment as simply being a form of a pseudo-hiding, disguising, and dissembling. Heidegger calls this the “one mode of concealment that for the Greeks…has codetermined the truth, the unconcealment and unhiddenness, of all beings.”^35^ This might be thought as a kind of overarching concealment (concealment 1) within truth itself.

Yet a second kind of concealment (concealment 2) concerns reflection upon its various modes (Weisen) and kinds (Arten) in its particularity. Heidegger classifies concealment 2 under two overall sorts: those that “displace” (2.a) and those that “shelter or “save” (2.b). Under a displacement concealment (2.a) fall the forms of setting aside, disappearing, putting away, being absent, having been destroyed, or withdrawing. Under the latter, a sheltering/saving concealment (2.b) fall safeguarding, preserving, and “rarifying,” namely, of that which is infinitely “rich” or to be treasured. There are forms of concealment that specifically pertain more to the domain of “presence” than of “absence,” and can be given to phenomenological description. Yet their being described can take place only through various forms of renouncement (e.g. resistance, ignorance). Further, there is never a full “unconcealment” of concealment, and the thing itself “reveals” its strata only inconspicuously. Intuition (Anschauung) never brings such phenomena to total “appearance.”

These forms of a sheltering/preserving concealment (2.b) are closer to the heart of “mysteriousness,” which essentially is far more complex than “the unexplained” or “unknown.” Un-knowing occurs in various forms of concealment and their many possible combinations: In experiencing mystery, one encounters an active concealing movement (Verborgenheit). There are various kinds of concealment at work within the particular experience of the mysterious, which might point to an “open secret” [offene Geheimnis] that is characterized by the fact that one knows that there is a secret, and one is familiar with it as a secret, yet one does not “know” the secret itself, as its most essential features are not “presenced.” This points to one of the paradoxes of how concealment is the productive and operative element within unconcealment: Its various indications immediately give the general fact that something is concealed and not-revealed. One is given a kind of intuition or awareness of such mysteries, yet accessing their multivalent features remains unthinkable, though the mystery prods one to attempt to do so.

Such a notion of the “mysterious” is formative to Heidegger’s thinking, and inconspicuousness is referred to as a character trait of its activities. It is concealment 2 under which is referenced the mysterious–an inherent movement within ἀλήθεια. Not unlike the former, concealment 1, the concealment at work within/as mystery should not be taken merely as a form of partially hiding or deception. In the Parmenides seminars, Unscheinbarkeit appears as one of the characterizations of concealment, especially as seemingly ordinary things manifest themselves mysteriously.^36^ Without being hidden, the mysterious remains inherently foreign, exceeding both calculability and inexplicability, and are thereby “characterized” by their own non-dialectical “category,” Unscheinbarkeit:

The mystery thus becomes a ‘residue’ still remaining to be explained. But since technical explaining and explicability provide the criterion for what can claim to be real, the inexplicable residue left over becomes the superfluous [i.e., the mysterious must exceed the explicable]. In this way the mysterious is only what is left over, what is not yet accounted for and incorporated within the circuit of explicative procedures. It would surely be simplistic and not thoughtful at all if we were saying that the little ego of some individual man were capable of elevating calculability to the rank of the measure of the reality of the real. Instead, the modern age corresponds to the metaphysical depth of the course of its history, when, in accordance with its will toward the unconditional ‘residuelessness’ [Restlosigkeit] of all procedure and all organizing, it builds broad avenues through all continents and so no longer has a place free from that residue in which the mystery would still glimmer in the form of mere inexplicability. The secret in the mystery [Das Geheime des Geheimnisvollen] is a kind of concealment [Verbergung], characterized by its insignificance [Unscheinbarkeit, i.e., inconspicuousness] in virtue of which the mystery is an open one.^37^

How naïve that man thinks his “little ego” [kleiner Egoismus] is capable of assessing the full measure of reality in a way that nothing is overlooked? The “secret,” which is a kind or sort of “covering over” [Verbergung, lethe] is in the mysterious. What is of most interest here is that the nature of a secret is most characterized by its Unscheinbarkeit; that is, inconspicuousness is the secret’s most distinctive and defining feature. Further, inconspicuousness is the definitive reason for why “mystery” maintains the status of remaining “open”—congealing into neither the visible nor invisible.

This version of Verbergung is the more passive version of covering in the sense of something already having the status of being covered-over. The root of which relates intimately with the more active “concealing” of Verborgenheit. It is in this sense that inconspicuousness plays the active role of “opening” (which is essential to Da-sein’s status as a being-open) the mysterious and keeping it open. The secret within mystery (which is the closest we get to any “opposite” of un-covering) is that, in some inconspicuous way, it remains open:

Another kind of concealment [the passive Verbergung] within the mysterious is displayed by the clandestine, under the cover of which, e.g., a conspiracy simmers [Verschwörung bewegt]. There the concealment has the character of an extended yet at the same time tightly knit ambush, lying in wait for the moment of the sudden outburst. The inconspicuous [das Unscheinbare] is here too. But now it [i.e., the inconspicuous] takes the form of camouflage and deception. Therefore this inconspicuousness [Unscheinbarkeit] must explicitly protrude [or trespass] everywhere and must always be concerned with safeguarding its outward appearance [or “shininess,” Scheins].^38^

Although deception does not characterize mysteriousness, there is also a kind of concealment within the mysterious that functions deceptively, and the inconspicuous also plays an active role in it, taking the form of a “camouflage.” That is not to say that it gives itself with/via another appearance, but only that it blends in with what is easily seen. When the inconspicuous takes this form, it trespasses (as it “protrudes” or hervortreten) into the field of visibility, yet it actively safeguards itself from being located. It can draw notice to its nature as non-disclosed. We once again see how the inconspicuous takes on a form distinct from the merely not-yet-known.^39^

Another operative role played by inconspicuousness within the mysterious is that it characterizes the protrusion of mystery into the open without “shining” (Scheins). The inconspicuous can work within the forms of both concealment and deception, without being reducible to either. Another way it is distinct from a straightforward hiddeness is that what is hidden is capable of being eventually uncovered or unconcealed if one makes certain efforts, while what is inconspicuous retains layers of unnoticability. What is inconspicuous is incapable of being exhausted, yet remains entirely immanent.

There is another “sort” of mystery to which Heidegger refers in the Parmenides Seminars–the uncanny (Unheimlich), which operates with an “astounding” inconspicuousness by being revealed/concealed in the ordinary. The “Divine for the Greeks,” as Heidegger claimed, is “the uncanny in the ordinary, the normal, the everyday.”^40^ The Uncanny is ever present yet not subjectable to a stable presence. The Uncanny represents the heart of concealment/unconcealment, and therefore is essential to the truth of Being. Like mysteriousness, the uncanny is characterized by its Unscheinbarkeit:

The uncanny is also not what has never yet been present; it is what comes into presence always already and in advance prior to all ‘uncanniness.’ The uncanny, as the being that shines into everything ordinary, i.e., into beings, and that in its shining often grazes beings like the shadow of a cloud silently passing, has nothing in common with the monstrous or the alarming. The uncanny is the simple, the insignificant [unscheinbar, i.e. inconspicuous], ungraspable by the fangs of the will, withdrawing itself from all artifices of calculation, because it surpasses all planning. The astounding for the Greeks is the simple, the insignificant [unscheinbar], Being itself. The astounding, visible in the astonishing, is the uncanny, and it pertains so immediately to the ordinary that it can never be explained on the basis of the ordinary.^41^

This can be likened to other comments regarding unconcealment in Poetry, Language, Thought:

We believe we are at home in the immediate circle of beings. That which is, is familiar, reliable, ordinary. Nevertheless, the clearing is pervaded by a constant concealment in the double form of refusal and dissembling. At bottom, the ordinary is not ordinary; it is extra-ordinary, uncanny. The nature of truth, that is, of unconcealedness, is dominated throughout by a denial. Yet this denial is not a defect or a fault, as though truth were an unalloyed unconcealedness that has rid itself of everything concealed. If truth could accomplish this, it would no longer be itself. This denial in the form of a double concealment belongs to the nature of truth as unconcealedness. Truth, in its nature, is un-truth.^42^

Both the ordinary, which simply and immediately “is,” and the uncanny, which does the work of “astonishing,” are primordially sutured to, yet capable of suspending one another’s activities in, the untruth/truth oscillation. This is one reason why the simple and everyday take on such prominent roles–they mark the ways in which the Greek gods are manifested (though without straightforwardly showing). For our purposes, it is helpful to highlight how the inconspicuous is a kind of uncanniness. The uncanny does not simply appear and then withdraw, but it does so in a way that subverts our entire knowledge and understanding of visibility and how appearance works: The uncanny “itself in its essence is the inconspicuous, the simple, the insignificant, which nevertheless shines in all beings.”^43^ However surprising, the inconspicuous “nevertheless,” despite its having the trait (Zug) to “not shine,” gives itself in all beings. It is this peculiar ability that makes what is familiar and ordinary to appear in an unimpressive and homely manner. It is this initial unimpressiveness of the uncanny that makes it so shocking and astounding beyond expectation. By merit of its inconspicuousness, the uncanny makes the entire world appear out of sorts.

The uncanny gives itself according to its own terms, and does so in all beings (this lends support to the thesis that Heidegger’s “phenomenology of the inconspicuous” should be applicable to his entire philosophical approach).^44^ And as an inconspicuous sort of concealment, its givenness is not explicable merely as “obvious” (selbstverständlich). It is likely that the “ordinary,” by merit of its familiarity and simplicity, is the best at concealing the mysterious precisely because it is inconspicuous. Much like the hyle of sense perception, one overlooks that which appears to be ordinary by merit of its lacking any profound ability to grab attention. The familiar and ordinary appears banal, and therefore the intelligibility of its phenomena is actively set aside in preference for seeing something else. The memory, overfamiliarity, and past experience with particular phenomena lends to their intelligibilities being-setting-aside from investigation, no matter the astonishing potentia they may hold. Meaningful presence so easily congeals into stativity.

## Forms of presence and the Zähringen seminar

This leads back to the Zähringen Seminar, wherein Heidegger argues that “presence presences” or presence gets presented (anwest nämlich anwesen) and does so in such a way that it’s active presencing can be bracketed momentarily and just long enough to disable the matrix of opposition between presence and absence. This is possible because the inconspicuous is (1) capable of being “indicated” (as it protrudes into the visible, hervortreten) or sketched (in distinction from indication as a straightforward “signaling” or “pointing”), and (2) radicalizes presence itself by forcing one through what is distant/absent (a kind of away-presence) in order to have an experience of what “is present” (a before-presence). The point at which this becomes “tautological thinking” is when awayness and beforeness are not easily distinguishable from one another. This is indeed an extension of Heidegger’s understanding of temporality.

The verb “to presence” effectively brings about or “presences” the present in both its temporal and spatial dimensions, which indicates that Heidegger has a certain “place” or space in mind when he makes this reference. In the appendix to the Four Seminars, Heidegger adds that the “location” in which presencing presences is “right ‘at’ and in unconcealment.” Presencing presences in the crevices of unconcealment, ἀλήθεια, which is not simply some “rigid openness” but one of vascillation, or of “encircling” that allows such presencing in what he calls a “…fitting, encircling revelation.”^45^ Such a revelation of presence tautologically presencing itself (which is characterized by the oscillatory movement between presence and absence) operates inconspicuously. One experiences the present as it is given, though such a being given is only of that which is always already there. Is this kind of tautology-speak mere sophistry, or is it indeed depicting a phenomenological reality?

Heidegger claims that this presencing of presence is “clearly a tautology,” and it is at this point that inconspicuousness becomes paramount:

We are here [at the aforementioned tautology] in the domain of the inconspicuous [Bereich des Unscheinbaren]: presencing itself presences. The name for what is addressed in this state of affairs is: to eon, which neither beings, nor simply being, but to eon: presencing: presencing itself. In this domain of the inapparent, however. ‘along this path there are a great number of indications.’^46^

Presencing is to be understood as a breaking into presence, which Heidegger claims gets at the very “untrembling heart of aletheia.” Thus, Being in this case is synonymous with truth, which manifests itself, and does so in the “domain” of the inconspicuous. There are “indications” that for Heidegger “must be understood in the domain of the inconspicuous,” and they are neither mere “signs” (for “indication must be understood here in the Greek sense: it is not something which stands as a sign for something else”), nor are they gestures to always accessible and unconcealed meanings. This demarcates the Greek understanding of truth: “Indication is what shows and lets be seen, in that it depicts what is to be seen.”

This kind of indication works in two senses. First, it actively depicts something in such a way that it is meaningfully present for the one to whom that which is given is depicted (e.g. a police officer sketching the image of a perpetrator of a crime for a victim). The depiction says something about one’s presently given context, yet such an indication is not meant to stand the test of time. And second, indication is that which gives some thing the opportunity to be itself, that is, to be what it always already is. In the thing’s being indicated, it comes forth or is partially unconcealed to the degree that its being indicated suffices to describe it. Our task is to attend to the present yet potentially shifting status of the thing and its phenomenality.

This leads us back to the very basis of Heidegger’s phenomenological project, as it gets distinguished from Husserl’s in the Zähringen Seminar. Although the tautology of presence getting presenced at first appears to be mere sophistry, tautology is the necessary phenomenological door through which one must enter in order to go beyond the central matrix of opposition between appearance/non-appearance. Tautology could here be thought as a kind of involution whereby an operation is inverted to determine that its inverse claim or operation is equal to it, though stated or arrived at differently. For Heidegger it was “Heraclitus [who] signified the first step towards” such a tautology, which leads to the most “primordial sense of phenomenology.”^47^ One seeks things in their provenance to grasp not simply that there are appearances (phainesthai), but how appearances ebb and flow in a way that they cannot be pitted against other phenomena. This is an “extreme” phenomenology because it reaches for a more primordial point of conception, but in a way that does not seek a static genesis. It is in this sense that “tautology is the only possibility for thinking what dialectic can only veil.”^48^ Necessary is a new attunement to the unconcealment of concealment (concealment 1) in general, and the various modes and forms of concealment in particular (concealment 2). We are not to attend to “presence” itself, but rather to the oscillation between presence and absence, which is marked by inconspicuousness.

Further, it is the non-appearing of phenomena that make for the possibilities of their appearing. This again can be exemplified in the sensical/categorial intuition distinction: When one has a fundamental experience with a particular horse, the hyle of that horse withdraws from experience and becomes inconspicuous despite its being the most physically or materially present: “It is the substantiality [the hyle, i.e.] that, in its non-appearance, enables what appears to appear. In this sense, one can even say that it is more apparent than what itself appears.”^49^ Enigmatically, what is overlooked is “more apparent,” and this tells us something about thinking in an inconspicuous key. In order actually to perform “an exercise in a phenomenology of the inconspicuous” though itself must be brought “into the clearing of the appearing of the inconspicuous.”^50^ This is that “domain” of inconspicuousness that Heidegger references in the Zähringen Seminar, and at the very least, one can conclude that Heidegger here is promoting a form of thinking that turns to what is inconspicuous. Yet provided its status as inconspicuous, any method of calculability will come up short in description. The inconspicuous is a domain of non-experience (which is distinct, e.g., from blind a priori conjecture) that is not graspable by pure volition.^51^ When inconspicuousness is given, it might give also, for example, a sense of ambiguity, disorientation, or confusion. Thus, when thought reaches the clearing (Lichtung) in which the inconspicuous is given, the content of what is thought undergoes a slippage between withdrawal/approach and unconcealment/concealment. This at least furnishes us with some broader features of Heidegger’s “phenomenology of the inconspicuous.”

## Three interpretations

These treatments of Heidegger’s usages of Unscheinbarkeit are by no means thorough, and likely are in need of further correction and engagement. There are a number of other places scattered throughout his work through which one might observe the word evolving into attaining the status of the concept that it reaches in his final seminar. Yet the interest of this paper has been to engage directly Heidegger’s own words about the topic so as to draw further distinctions hopefully in order to dispel some common misunderstandings (e.g. Janicaud’s seeming conflation between invisiblity/inconspicousness). Further, it also remains necessary to distinguish Heidegger’s approaches to Unscheinbarkeit from some recent constructive developments of the notion in contemporary phenomenology, such as Figal’s recent Unscheinbarkeit (2015), which explicates the role of space in phenomenal experience via intuition.

How, then might “eine Phänomenologie des unscheinbaren” be interpreted? I suggest there are at least three possible interpretations, which still retain room for further distinction and clarification. (1) The first possibility is the most likely to be accepted by readers of Heidegger due to its generality: Unscheinbarkeit is a character trait that any and all phenomena are capable of enacting, and therefore inherent to a Heideggerian phenomenology. The phenomenological process is an interminable one, and Being announces itself in and through the various strata of phenomena. Further, the categorial/sensical distinction is applicable to the intuitive grasping of any phenomena (not specifically inconspicuous phenomena). Such an interpretation would entail that the employment of phenomenology would be for the sake of greater experiencing “the covered” (Λήθη) by determining which of its many forms (Arten) are employed through the performance of a kind of reduction. Appropriation (Ereignis) is the moment at which the inconspicuousness of phenomena becomes ever “present” to Dasein as he/she is appropriated (ereignete Dasein). One is aware that things are coming into appearance, yet the intricacies of the possible combinations of their phenomenal data are inevitably obscure (though they “shine” in the ordinary). Inconspicuousness resides within any phenomenal appearance, and the task (of a lifetime) would be to make Being intelligible. Although true, this approach is irreducible to the notion that all phenomena bear layers of intelligibility that can be accessed via phenomenological description. Inconspicuousness does not concern the not-yet-appearing of phenomena, but how “the clearing” is itself a fundamental “concealment” that is productive.

Most secondary scholarship on this notion of Heidegger’s interpret it more or less in this way. Dastur claims the inconspicuous to demarcate “the nonappearance that resides in all appearing, the event itself of apparition and the giving of being.”^52^ Any appearance retains the potential for Being to be given in an event, and inconspicuousness is intertwined within appearing Taminiaux (who played a role in the development and publication of the Zähringen seminar) seems to hold to a similar thesis, concluding that the coming-into-appearance of things is always transfixed with an inconspicuous and unforeseeable “excessiveness at the very heart of seeing.”^53^ The inconspicuous subsequently can “never be brought into view,” as Gonzalez interprets, for it remains instrumental to the mysteries of being.^54^ Benson and Simmons believe that “Phenomenology, at its root, is for Heidegger an inquiry into appearance in such a radical way that it begins with a receptivity to the inapparent insofar as it allows for the apparent to then be considered according to the categories of being.”^55^ Then there is Figal, who suggests that phenomena can “only be adequately understood in their unison with the inconspicuous.”^56^ These interpretations remain rather consistent, and it seems the majority of scholars would subscribe to this first interpretation, which holds that all phenomena no matter how “ordinary” or ontic, have within them the paradox and radical potentia of revealing Being inconspicuously.

Many would subscribe to this view because it fits quite nicely into how we generally interpret Heidegger more broadly on phenomenal experience. It is surely the case that the a priori of appearance can never be brought into manifestation fully, and that there is some inner oscillation at work between unconcealment and concealment. Being transcends the entities of that which it “shines” in and through. After all, the Zähringen Seminar was inspired by Jean Beaufret’s question concerning the Seinsfrage and the way in which the transcendens of Being is phenomenology’s most primal quest.^57^ Yet one complication with this view concerns the way in which phenomenology therefore is to be reconstructed. If phenomenology, in every instance must contain some element of inconspicuousness, then therefore is not one obliged to seek means of attunement to the specific features and activities of inconspicuousness in every attempt to think phenomenologically? In referring to a phenomenology of the inconspicuous, this “of” indicates either that the method is applied to what remains inconspicuous (as we will study below) or it demarcates this method itself, and how it turns to the appearance/non-appearance of things.

Following through with the former thesis, that such inconspicuousness is to be integrated into Heidegger’s overall approach to describing the intelligibility of things, then it would be necessary to seek out ways to account for such inconspicuousness, otherwise the phenomenological approach would remain always incomplete. It is precisely because of the generality of this interpretation that it would therefore call for a cursory re-assessment of Heidegger’s entire approach: To what degree does Being itself, as a non-metaphysical mystery, go misunderstood if its inconspicuousness is not brought under consideration? To what degree is be-ing, the coming to be or giving of the being of beings, “intrinsically unapparent,” as Polt suggests to be especially true of the later Heidegger?^58^ It is in Heidegger’s later work that the coming-to-be of Being takes on a more mysteriously co-present concealing as one is thrown into, or appropriated within the world.^59^ Although concealment is inherent within this approach to thinking, mysteriousness must remain inconspicuous in order to not fall back in the quagmire of the ontotheological constitution of metaphysics, which Heidegger repeatedly warns about. Despite the potential saliency of these concerns, it is very likely the case that inconspicuousness can be interpreted as a characterization of Being, in general. Nevertheless, this view appears to not account for why in the Zähringen Seminar a “phenomenology of” the inconspicuous is referenced, in which one engages through some kind of “exercises.”

(2) A second interpretation of Heidegger’s phenomenology of the inconspicuous is a bit less demanding to rethink Heidegger scholarship. In this case it is not so much that all of phenomenology is to be rethought as a “phenomenology of the inconspicuous,” per se, but that such a phenomenology is a species within the genus of phenomenology as the particular study of the stratification inconspicuousness within experience. Heidegger indeed in the Zähringen Seminar is introducing something different, what Janicaud wagered to be a “new form of thought.”^60^ In which case, a new, particular step is being introduced into all of phenomenology that involves ones turning attention to the various modes of potential hiddenness (Verborgenheit and its cognates) within all phenomena, and “inconspicuousness” is now to be included as a form, mode, or “manifestation” among them. In which case, one can only get at these particularly “dark” or obscure corners of experience within intuition through actually performing the “exercises” of such a phenomenology. How, if at all possible, might one access “inconspicuousness” in and of itself?

It may be that such exercises are meant to train one on getting involved in the description of the aforementioned oscillation between the present/absent. Such an exercise would be like a reduction to the inconspicuous withdrawal of phenomena. There are, for example many things that are inconspicuous to me right now: the hyle of the plastic keyboard upon which I type; the sensation of an ache in my back to which I have grown so accustomed in the last hour. I exercise my ability to experience things and be affected by them despite their ordinary status of being accepted into my meaning-given experience within the world. By turning attention to the pain in my back, something new might be revealed to me, such as my human contingency. By relaying to the physical experience of typing I might reflectively engage in a better understanding the relation that is being formed with my operating system, which typing mediates. This remains consistent with the notion that the the uncanny is most expressed in ordinary things. As I engage what has become inconspicuous to me, then like a rack and pinion, the most profound potentia of phenomena are given warrant (schein) to make an appearance, despite the fact that they may only give themselves contingently. It cannot be overstated: unscheinbar is not a cognate of unsichtbar, or “invisible,” and it is a phenomenon that enacts the unique ability to resist being conjured within the present. Under this interpretation, all phenomena, no matter how ordinary, are capable of bearing such inconspicuousness, yet one engages it only at a particular step or time within phenomenological reflection.

(3) The third interpretation of Heidegger’s unscheinbar would hold that it is a direct reference to specific, unique, and distinct phenomena that paradoxically exceed the visible/invisible (sichtbar/unsichtbar) polarity, yet still somehow are present and affective. If this is the case, then there should also be a corresponding approach to “accessing” the very special means of phenomenality such things hold, which might be studied only when one is trained on the modalities of inconspicuousness (e.g. forms of hiding, which can be thought as the unique unconcealment or manifestation of inconspiciousness). They are very particular sorts of phenomena, which require a very particular type of phenomenology that corresponds with their unique modes of manifestation. They are phenomena that forfeit their phenomenality in a unique way, yet still remain qualifiable as fully, present-at-hand phenomena. There are intrinsically inconspicuous phenomena that do not reach presence in the same way that “ordinary” object-beings can.

Though they may appear in the ordinary, or at least, present themselves as if they are ordinary (as one might glean from the Parmenides Seminars), it may be that not all ordinary phenomena have the potential for such inconspicuousness. They are phenomena that follow Heidegger’s amendment to the constitution of phenomenology, Husserl’s “principle of principles,” which trains one on the making present of that which is hidden, or the direct description of what clearly appears to oneself in consciousness.^61^ The possibility of Inconspicuous phenomena, which could be understood to operate in the margins or residue of what gets bracketed in Husserl’s reductions, calls for a phenomenology for thinking about them and making them intelligible. The same categorial/sensical intuition distinction still remains, yet now at full throttle, and in reference to specific phenomena that have the potential to more profoundly alter one’s experience. It would be through thinking–tautologically–the presencing (das Anwesen) of presence (die Anwesenheit), that one might arrive at an experience of these phenomena.

Two thinkers who seem to hold to the view that there are unique phenomena that are inconspicuous are Janicaud and Marion. For Janicaud, who insists that “[t]his ‘phenomenology of the inapparent’ is not reducible to a mere appendix to the thought of the later Heidegger” thinks that such an approach is meant “to train sight and hearing to get as close as possible to phenomenality. [And therefore] the ‘phenomenology of the inapparent’ is a phenomenology of proximity.”^62^ It concerns proximity because it is a matter of the presencing of presence and the closeness/farness interaction always taking place in the grand experience of Being. This tautology is about returning to “the first self-evident insight of phenomenal appearing: time temporalizes, saying speaks, the world worlds.”^63^ One engaged in such a phenomenology actively seeks out sameness in the world by attending to “the withdrawal of things.”^64^ Yet most importantly, Janicaud’s interpretation of this concept becomes clearer in his earlier book Chronos, in which he claims “the inapparent is that which escapes common experience, that which does not appear of itself at first glance” and is a phenomenality par excellence.^65^ There are phenomena that have the unique quality or state of being inconspicuous and of not attracting attention. Despite Janicaud’s occasionally appearing to conflate the terms inapparent/invisible, he nevertheless refers to them as quite specific phenomena in Heidegger’s approach. Janicaud’s central critique, after all, was that those thinkers associated with the pejoratively named “theological turn” in French Phenomenology relied on Heidegger’s approach to the “inapparent.”

One of the thinkers associated with precisely such a turn in French thought was Jean-Luc Marion, for whom Heidegger’s Being, as early as Sein und Zeit, is intrinsically “inapparent” or inconspicuous. Being is unique insofar as it does not present itself like other phenomena; nevertheless, Being is a phenomenon despite not appearing ontically.^66^ There are unique phenomena that appear inconspicuously, and “this [is a] paradox–a phenomenology of the unapparent as such, and not simply of the not-yet-appearing.”^67^ Marion, in his now famous “reduction to givenness” (“so much reduction, so much givenness”) attempts to show, like Heidegger, the inexhaustible surplus or excess of phenomena. Yet for Marion, some are more “saturated” or ripe with possibility than others, which are “poor” in saturation. Then there are phenomena that, when appearing to be “lacking” in phenomenal data, “shine” all the more by their absence.

An argument could be made that for Heidegger there are phenomena that might carry traits of inconspicuousness more than others, such as those that he claims to house the mysterious. In “On the Essence of Truth” das Geheimnis is claimed to be the self-concealing nature of Being and truth.^68^ Then, in the Introduction to Metaphysics, Heidegger argues that the origin of language and its structure “remains a mystery[;]” for language “can have begun only from the overwhelming and the uncanny, in the breakaway of humanity into being.”^69^ And finally, the essence of humanity (Das Wesen des Menschseins) is, or gives itself in mysteries or secrets.^70^ Since we already know from the Parmenides Seminars that Unscheinbarkeit is the prime characteristic of the mysterious, then it is brought to bear upon these aforementioned particular phenomena that house the mysterious.

This latter interpretation of Heidegger’s phenomenology of the inconspicuous is not without concern, however: If these phenomena are to remain inconspicuous, then how might they ever be accessed or described? Would not a direct explanation of them exhaust them of their inconspicuousness? Is an aspect of inconspicuousness sufficient for one to have had an experience with that which is presented to experience as inconspicuous? One wonders if there could be an approach to such inconspicuousness without it ultimately undermining its own characteristics. It also is concerning to think that such a phenomenology of the inconspicuous could be claimed as a new point of access to a metaphysical eidos; again, one that Heidegger already banished in his critique of ontotheology. A phenomenology of the inconspicuous must be a study of Being in this world, and not employed to justify a return to the metaphysics of presence. What is inconspicuous must remain as such, despite its own inner tensions.

## The inconspicuousness of Heidegger

There likely are reasons why Heidegger, after his last seminar in 1973, left his treatment of inconspicuousness slightly ambiguous. Although each of the aforementioned interpretations reflect some interest of Heidegger’s, it is a version of this third interpretation that I find the most convincing as well as the most fecund for phenomenological thinking. Aside from the fact that the later thesis reflects a slightly more radical and daring attempt (which I find to be most likely, given that it was introduced in his last seminar) for phenomenology, at the very least, it might be suggested that there are phenomena that have a greater tendency to inconspicuousness than others. There are likely varying shades of inconspicuousness that phenomena can bear, and those shades tell us more about ourselves–what we care about, how we ignore, disguise, and select data–than they do about the phenomena. While there are indeed various means by which all phenomena, following the categorial/sensical intuition distinction, must remain to some degree generally inconspicuous within phenomenal experience, it seems some things have the tendency to become inconspicuous because they instantiate the right carburation between uncanniness and ordinariness. Even if all ordinary phenomena are capable of bearing, sending, or presenting uncanny mysteriousness, some phenomena will take on more profound layers of intelligibility as I am the one to make them meaningful and select them from out of this world’s steady and constant stream of possible intelligible data.

Overall, an approach that allows for the experience of something inconspicuous involves the active being-caught-up in the ordinary in such a way that something mysterious can shine through it. The inconspicuous is in the margins of what typically gets reduced to be present, and therefore it represents an always standing “residue” that Heidegger seems to surmise to be bracketable, despite being between the cracks of presence and absence. This is why one means of truth’s truthing and one’s realization of what is inconspicuous seem to become one in the same. This is the phenomenological seeing (as stated in the Zahringen Seminar) at which one might arrive, which is a contested presencing that the will could never apprehend or grasp. What is inconspicuous amounts to the most basic transgression of how we typically understand phenomenal appearance.

Ultimately, a phenomenology of the inconspicuous should matter to us because it aims to take seriously what the sharp distinctions between appearance/non-appearance have generally sought to veil through their being cast in various matrixes of opposition. How we take it that certain things are no longer worth our attention, how we take people and family for granted, and how we overlook the least of those around us by merit of “shinier” things, celebrities, or spectacles that command our attention, all provide some reasoning for why this topic is an important one. Overwhelming privilege is accorded today to whatever can present the greatest possible degree of unconcealment: The greater degree the spectacle, we are complicit to believe, the more sacred an event becomes and the closer to divinity it presents itself. This bears consequences for what has become most familiar, and therefore insignificant to us. Yet with a bit of optimism, phenomenology may provide tools for sharpening our ability to take seriously again what is ordinary and familiar. And again, as Heidegger over and again taught, the Uncanny (Unheimlich) “itself in its essence is the inconspicuous, the simple, the insignificant, which nevertheless shines in all beings.”^71^ Sometimes phenomenology, which often has the reputation for obsessing over the abstract minutia and detail of things, should instead be used to address ordinary things. Freud was serious when he once quipped that “sometimes a cigar is just a cigar,” and it may very well be such a seemingly insignificant thing as the cigar that marks the “saving power” for humanity today.

## Footnotes

1 An etymology of “conspicuous” also leads to the word scope, which refers to an aim or purpose of the seen, from the root spek, or observe. This is where skeptesthai finds its rooting in “to look at” or investigate.

2 I choose “inconspicuous” because in English it insinuates that something is fully present to us, yet consciously we are unaware of its specific features in a given moment. Yet there is certainly no consensus on how unscheinbar is to be translated into English. Figal recently shares a similar sensitivity to my own, and translates it as “inconspicuous.” Most of the French choose “inapparent” likely because it at first appears to be metonym of the French “l’inapparent.” Then, there is William McNeill’s strange reference to unscheinbar as “improbable” (though it does not get translated as such in Pathmarks): “We are held enraptured by something ‘improbable’ (unscheinbar), by something that, in its shining forth, also does not shine, or does not yet shine–by the approach of an event that could not have been foreseen.” McNeill (1999, p. 291).

3 Heidegger (1986, pp. 372–407).

4 What Heidegger names a tautology, however, is not void of conflict, but is indicative of a deep tension out of which “differentiation” might be given.

5 Heidegger’s “phenomenology of the unapparent,” asserts Janiciaud, does not “lead us away from the question of the theological turn. But on the contrary, it places us at the crux of the matter where everything is decided: at the point of rupture between a positive phenomenological project and the displacement of its ‘possibility’ toward the originary [originaire]. What troubles some can gratify others. If the ‘phenomenology of the unapparent’ finally makes all rule-based presentation of the phenomena vacillate in favor of a hearkening to a world whorled with silence, here–against all expectations–is a line extended toward the originary, the nonvisible, the reserved. Ready to renounce a thematic phenomenology, the candidates to the theological heritage will content themselves with a phenomenology of points and dots.” Furthering the critique, Janicaud suggests that “[i]f the ‘phenomenology of the unapparent’ is to be interpreted not as a regression, but as full of promise, then the most audacious soundings stand permitted.” Janicaud (2005, pp. 30–31, cf 28–29).

6 In a subsection of his book Chronos entitled “La tentative d’une ‘phénoménologie de l’inapparent’” Janicaud suggests that “The formula ‘phenomenology of the inapparent,’ which is both paradoxical and enigmatic, seems to complicate the problem instead of clarifying it.” (“la formule ‘phénoménologie de l’inapparent’, à la fois paradoxale et sibylline, paraît compliquer le problème au lieu de l’éclairer.”) Janicaud in general concludes that these comments of Heidegger’s in the Zähringen Seminar serve to throw more decisive light on the radical change that Heidegger wished to have upon the theory of time. Janicaud (1997, pp. 157–58).

7 Janicaud (2005, p. 73). Janicaud explicitly refers to the invisible as inapparent: “It will no longer be a matter of suspecting a return to the ‘theological’ for the phenomenologies of the invisible (Inapparent), of the Other, of auto-revelation, or of pure givenness, but rather to ponder if phenomenology must not be radically atheistic in order to succeed in its project to attain, describe, and speak of the ‘thing itself.” (Ibid., p. 9). Much later in this text, Janicaud argues that such an approach can be conceived as a “minimal phenomenology:” “This ‘phenomenology of the inapparent’ is not reducible to a mere appendix to the thought of the later Heidegger. If it means something, and if Heidegger was not simply toying with his audience, it is in fact the inauguration of a new meditative form of thought. The ‘practical studies’ of the later Heidegger (like the exercises in Daseinanalyse from the Zollikoner seminar) are undertaken in this vein. To call this unusual phenomenology ‘minimal’ is not to belittle it. Rather it is to give the fullest sense to this very difficult attempt to train sight and hearing to get as close as possible to phenomenality. The ‘phenomenology of the inapparent’ is a phenomenology of proximity” (Ibid., p. 75).

8 Even in the original Greek conception of truth, its nature of “non-shining” was overlooked, focusing instead on things’ radiance, according to Heidegger: “The Greeks were the first to experience and think of phainomena as phenomenon. But in that experience it is thoroughly alien to the Greeks to press present being into an opposing objectness; phainesthai means to them that a being assumes its radiance, and in that radiance it appears. Thus appearance is still the basic trait of the presence of all present beings, as they rise into unconcealment.” Heidegger (1985, p. 132, 1971a, p. 38).

9 Taminiaux, Dastur, and Marion, among others, have engaged directly, albeit briefly, this topic in Heidegger’s work.

10 For Figal, “Raum, so last diese Überlegung sich zusammenfassen, erscheint nicht, sondern ist unscheinbar, so das eine Phänomenologie der Äußerlichkeit, eine realistische Phänomenologie, also eine Phänomenologie der Unscheinbarkeit ist.” Or “Space, to sum it all up, does not appear, but is inconspicuous, thus the phenomenology of externality, which is a realistic phenomenology, is a phenomenology of the inconspicuous.” This book follows Figal’s recent resignation from the role of President at the German Heidegger Society, Figal (2015, p. 4).

11 Figal (2015, p. 11). “Dass es Phänomenologie, die von Unscheinbaren her denkt, bisher noch nicht gab, könnte eine voreilige Behauptung sein, denn in jedem Fall gibt es einen auf sie hindeutenden Titel. Heidegger hat ihn geprägt; die spätesten Zeugniss seines Denkens dokumentieren ihn.” Or, “[t]he claim that there has not yet been any thinking of a phenomenology of the inconspicuous would be a hasty assertion, because the title has definitely been already suggested. Heidegger has crafted it; the latest testimony of his thinking documents it.”

12 Figal (2015, p. 15). Figal uses this claim to introduce how his approach is to be distinguished from Heidegger, futher asking: “Ist Heideggers tautologisches Denken, wie es skizziert und aus seiner Entwicklungsgeschichte erläutert wurde, wirklich eine Phänomenologie des Unscheinbaren? Wenn das tautologische Denken so zu verstehen ist, wie es dargestellt wurde, darf die Frag emit ‘nein’ beantwortet warden.” Or “[i]s Heidegger tautological thinking, as outlined and explained in its developmental history, truly a phenomenology of the inconspicuous? If his tautological thinking is to be understood in its presentation, the question may merit the answer ‘no.’”

13 It is also of note that the term “away” is associable with Weg or “path.” The Holzweg, or hiking trail that has no clear or apparent path because the needles or leaves cover the forest floor, and therefore one must be aware of the environment of the path (such as light, markers, scents, etc.). As Pöggeler suggested, Weg characterized perfectly Heidegger’s meandering or wandering approach to thinking. See Pöggeler (1991).

14 See Martin Heidegger (1987, p. 325, 248). Sheehan interprets the “da” of dasein to suggest that the human occurs as the “da,” that is to say, the “out thereness” or clearing of being. For Sheehan, “[i]n order to emphasize the unique characteristic that Heidegger intends to bring out by both Dasein (existentiel) and Da-sein/Existenz (existential), I will translate all three of those terms as “ex-sistence,” hyphenated to stress its etymology.” In part, this is because “Heidegger would have us hear the Latin ex + sistere, where the “ex-” or “out- and-beyond” dimension of human being forms an openness or clearing that he called “the Da” Sheehan, (2014, p. xvi).

15 In a most recent translation of Heidegger’s works (2015) is another engagement with Parmenides concerning time, which in its all-powerful and incalculable essence, is responsible for allowing everything to manifest itself that is hidden; and to hide whatever has appeared. For Heidegger, Parmenides was gesturing, in his philosophy of time, to how Being relates to presence in a significant way, yet is not immediately distinguishable from “absence,” which gets incorporated by presence.

16 For Dreyfus “Heidegger holds that we must learn to appreciate marginal practices – what Heidegger calls the saving power of insignificant things–practices such as friendship, backpacking in the wilderness, and drinking the local wine with friends. All these practices remain marginal precisely because they resist efficiency” Dreyfus (1993, p. 310).

17 Heidegger (2003, p. 80). “So verstanden ist die Phänomenologie ein Weg, der hinführt vor…und sich das zeigen läßt, wover er geführt wird. Diese Phänomenologie ist eine Phänomenologie des Unscheinbaren.” Heidegger (1986, p. 377).

18 Heidegger (2003, p. 80; 1986, p. 377).

19 “And yet—beyond what is, not away from it but before it, there is still something else that happens. In the midst of beings as a whole an open place occurs. There is a clearing, a lighting [Lichtung]. Thought of in reference to what is, to beings, this clearing is in a greater degree than are beings. This open center is therefore not surrounded by what is; rather, the lighting center itself encircles all that is, like the Nothing which we scarcely know. That which is can only be, as a being, if it stands within and stands out within what is lighted in this clearing. Only this clearing grants and guarantees to us humans a passage to those beings that we ourselves are not, and access to the being that we ourselves are. Thanks to this clearing, beings are unconcealed in certain changing degrees. And yet a being can be concealed, too, only within the sphere of what is lighted. Each being we encounter and which encounters us keeps to this curious opposition of presence in that it always withholds itself at the same time in a concealedness. The clearing in which beings stand is in itself at the same time concealment. Concealment, however, prevails in the midst of beings in a twofold way.” Heidegger (1971b, p 51).

20 Heidegger (1962, p. 219; 1977a, p. 262).

21 Sheehan interprets that“[f]or us, the Sein of something shows up only in discursive thinking and acting—that is, only when we take a thing as such-and-so, or in terms of this or that possibility. When I take something as, whether in theory or praxis, I understand the Sein of the thing, whether correctly or incorrectly.” Sheehan (2014, p. 21).

22 Heidegger (1962, p. 29).

23 Heidegger (1962, p. 53; 1977a, p. 30).

24 Heidegger (2003, p. 70).

25 A number of questions remain, however, for whether or not the away/presence structure is tenable beyond how we are to think of “static structures” in its more classical and Husserlian sense. Steinbock would hold that “in many if not most cases, static structures are surpassed through ‘deeper’ genetic analyses, and genetic matters and methods are ‘rattled’ or ‘ruined’ by generative ones.” The home/away, the “homeworld/alienworld” structure may be articulated according to Steinbock’s provisional/transcendental relation in the life world. Steinbock (1995, p. 265).

26 This special sort of “seeing” cannot be attained through the “reading of books,” claims Heidegger here. Heidegger (2003, p. 89).

27 Heidegger (1968, p. 9). There Heidegger also refers to the nature of the beauty artwork gaining its source from the truth of being Inconspicuous: “Beauty is a fateful gift of the essence of truth, whereby truth means the unconcealment of the self-concealing. The beautiful is not what pleases, but what falls within that fateful gift of truth which comes into its own when that which is eternally unapparent [unscheinbare, i.e., inconspicuous] and therefore invisible attains its most radiantly apparent appearance.” Heidegger (1968, p. 19).

28 Heidegger (1971b, p. 31).

29 Heidegger (1985, p. 247) claims that “Das Ereignis ist das Unscheinbarste des Unscheinbaren” or “appropriation is the most inconspicuous of the inconspicuous.”

30 It is in this sort of concealment that Heidegger begins to gesture in his meditations on technology; it operates “in the horizon of scientific and technical discoveries.” And “when the concealed in this sense is brought into unconcealedness, there arise ‘the miracles of technology’ and what is specifically ‘American.’” Heidegger (1992, p. 64).

31 See here John Caputo’s claim that A-lethia is no longer a Greek word for Heidegger. Caputo (1993, p. 21).

32 Heidegger (1992, pp. 64–65).

33 Heidegger (1992, p. 14). There are different types of closure, and different ways of interpreting unconcealment. Unconcealment “can mean concealedness is taken away, cancelled, evicted, or banned, where taking away, cancelling evicting, and banning are essentially distinct.”

34 Heidegger (1992, p. 62).

35 Heidegger (1992, p. 62).

36 For Rudolf Bernet, such phenomena would include those “of oblivion, anamnesis, the rare, the gift and the secret.” As for the secret, “it can only be saved if one renounces using the mysterious thing for one’s personal profit. In order to keep the secret of this thing, one must, Heidegger says, keep silent. Only silence preserves the secret, only silence respects the simplicity and the Unscheinbarkeit with which the secret offers a glimpse of itself through a thing which, by withdrawing for the sake of its mystery, runs the risk of going unnoticed.” Bernet employs Heidegger’s Unscheinbarkeit to better understand the secret: “The unconcealment of the concealment which is typical of the true secret, the coming into presence of what stays necessarily unapparent in the secret can only be realized under the form of an appearance that goes almost unnoticed or, as Heidegger says, that is characterized by its ‘‘insignificance’’ (Unscheinbarkeit)." Rudolf Bernet (2014, pp. 353–371).

37 Heidegger (1992, p. 63; 1982, p. 93).

38 Heidegger (1992, p. 63).

39 “Far away from these modes of concealment, and yet within the sphere of the same essence, resides the concealed in the sense of the merely not yet known” Heidegger (1992, p. 39).

40 Heidegger (1992, p. x).

41 Heidegger (1992, p. 101).

42 Martin Heidegger (1971b, p. 53).

43 Heidegger (1992, p. 105). The uncanny is given in the ordinary: “the uncanny, or the extraordinary, shines throughout the familiar ambit of the being we deal with and known, beings we call ordinary.”

44 Heidegger (1992, p. 106): “Yet what we are calling the ‘uncanny’ we still grasp on the basis of the ordinary. What the so-called uncanny is in itself and what first admits of the character of the uncanny as its consequence, that is based on the shining into beings, on self-presentation, in Greek: daio.” And then on p. 130: “And this original belonging together of both, precisely as primordial must also possess the inconspicuous character of what, like a source, comes to presence out of itself in its essence.”

45 Heidegger (2003, p. 96).

46 Heidegger (2003, p. 79).

47 Heidegger (2003, p. 80). Parmenides helps Heidegger point beyond the "dialectic" which "as is said in Being and Time, is ‘a genuine philosophic embarrassement’).” For Raffoul and Mitchell, the translators of this seminar, Heidegger’s naming phenomenology that of the “inapparent” is an adaptation of the “methodology in its most extreme possibility and formulation” Raffoul and Mitchell (2003 p. xvi).

48 Heidegger (2003, p. 81).

49 Heidegger (2003, p. 67).

50 Thought must be brought “into the clearing of the appearing of the unapparent.” Heidegger (1983, p. 115). Earlier, on April 16, 1973 he writes "[f]or me it is a matter of actually performing an exercise in a phenomenology of the inapparent; by the reading of books, no one ever arrives at phenomenological 'seeing'" Heidegger (2003, p. 89).

51 Heidegger (1992, p. 64). Regarding manifestation as unconcealment, “We are here only broaching a realm whose fullness of essence we hardly surmise and certainly do not fathom, for we are outside the mode of experience proper to it.”

52 Dastur (2002, p. 146). See also Miguel de Beistegui (2004, pp. 115–116, cf 127), as well as Gérard Guest (2002, p. 123).

53 For Taminiaux, this kind of seeing “grasps things in their coming-into-appearance, intentionality, is itself transfixed with excessiveness; seeing must be beyond the given in order for things to be and to be what they are.” Taminiaux (1977, p. 79).

54 Such inconspicuousness entails “a continually self-deconstructing attempt to bring into view what can never be brought into view but where the always inapparent could nevertheless be indicated in the very process of self-deconstruction.” Perhaps this concept is left ambiguous “precisely in order to be able to practice dialectic while insisting on and aspiring to something else which this very practice undermines.” Gonzalez (2011, p. 308). In SuZ Heidegger states the aims of phenomenology as clearly as possible: “And precisely because phenomena are initially and for the most part not given phenomenology is needed. Being covered up is the counter concept to “phenomenon.” Heidegger (1962, p. 31).

55 Benson and Simmons continue, for Heidegger “Phenomenology is not merely about phenomena, but ultimately about phenomenality itself.” Benson and Simmons (2013, p. 41).

56 Figal (2015, p. 11): “Wenn die bisher entwickelten Überlegungen zum Verhältnis von Erscheinung und Unscheinbarkeit überzeugend sind, wären Phänomene im skizzierten Phänomenologischen Sinne also nur angemessen in ihrer Zusammengehörigkeit mit dem Unscheinbaren zu verstehen.”

57 As Taminiaux put it: “Heidegger’s fascinated gaze found in the Logical Investigations the emergence of a group of themes which incite the Seinsfrage in Sein und Zeit: namely that being transcends the entity, that being is the transcendens par excellence, that being is in a special sense the phenomenon of phenomenology, that the entity’s coming-into-appearance requires a prior understanding of being, that this understanding, to the extent to which it is beyond, is nonetheless inseparable from an exposition of an entity, that the excessiveness of being is the cradle of truth.” Taminiaux (1977, p. 79).

58 Already in Sein und Zeit, according to Marion, Being is intrinsically inconspicuous because it relates differently with “presence” than do ontic phenomena. Yet Polt thinks that the Heidegger of Being and Time “claims that the being of beings is simply not-yet-appearing (being can be thematized as a phenomenon, even though it can never appear as ontically present, (SuZ 35); in contrast, the Contributions focus on be-ing (the giving of the being of beings) and claim that it is, at least in some ways, intrinsically unapparent.” For Polt, the later Heidegger seems to operate with a more inconspicuous “being” than the earlier one. Polt (2006, note 38).

59 For Heidegger “All revealing belongs within a harboring and a concealing. But that which frees–the mystery–is concealed and always concealing itself.” Further, “Freedom is that which conceals in a way that opens to light, in whose clearing shimmers the veil that hides the essential occurrence of all truth [i.e. a-lethia] and lets the veil appear as what veils.” Heidegger (1977b, p. 330).

60 Janicaud (2005, p. 75).

61 Another matter is that such an approach is still described according to possible phenomena for a subject, an "I" who is supposed to be the constitutor of the phenomenon. Manoussakis worries that “the world becomes a private spectacle for consciousness, a consciousness that is also the absolute director and the exclusive audience of this performance. Husserl himself was aware of the ‘grave objection’ that already arises here” Manoussakis (2004, p. 56).

62 Janicaud (2005, p. 75).

63 Janicaud (2005, p. 73).

64 Janicaud (2005, p. 75). “The ‘phenomenologist of the inapparent’ is no longer an ideal spectator of the truth of the world and of its essences: he learns to inhabit the world ‘at arm’s length’ from the withdrawal of things.”

65 In Chronos Janicaud refers to how “the inapparent is that which escapes common experience, that which does not appear of itself at first glance.” (“l’inapparent comme ce qui échappe à l’expérience la plus courante, ce qui n’apparaît pas de soi-même au premier regard.”) After briefly offering his own ways of interpreting this concept (that which can never be brought to light, that which disturbs phenomenology, and that which is the inherent nature of temporality) Janicaud seems to suggest that all three carry some weight, yet temporality remains a paramount concern: “Now what is the phenomenality par excellence that, neither immediate nor ontic, cannot be reduced to an eidetic aim? It is precisely the one that reserves the most problematic of ‘phenomenalities’: the temporal dimension.” (“Or quelle est la phénoménalité par excellence qui, ni immediate ni ontique, ne se laisse pas non plus réduire à une vise eidétique? C’est précisément celle qui reserve la plus problématique des “phénoménalités”: la dimension temporelle.”). Janicaud (1997, p. 159) See also Janicaud (2000, pp. 28–31; 2005, p. 100, note 14).

66 Marion (1998, p. 60).

67 Marion (1998, p. 60).

68 Mystery, das Geheimnis, refers to the self-concealing nature of Being. See Withy (2015, p. 133).

69 Heidegger (2014, p 182).

70 Ibid., p. 175.

71 Heidegger (1992, p. 105). It bears repeating that the uncanny reveals itself from within the ordinary: “the uncanny, or the extraordinary, shines throughout the familiar ambit of the being we deal with and know, beings we call ordinary.”
